# Efficient anti-Prelog enantioselective reduction of acetyltrimethylsilane to (*R*)-1-trimethylsilylethanol by immobilized *Candida parapsilosis* CCTCC M203011 cells in ionic liquid-based biphasic systems

**DOI:** 10.1186/1475-2859-11-108

**Published:** 2012-08-16

**Authors:** Bo-Bo Zhang, Jing Cheng, Wen-Yong Lou, Pan Wang, Min-Hua Zong

**Affiliations:** 1 State Key Laboratory of Pulp and Paper Engineering, South China University of Technology, Guangzhou, 510640, China; 2 Key Laboratory of Industrial Biotechnology, Ministry of Education, School of Biotechnology, Jiangnan University, Wuxi, 214122, China; 3 Laboratory of Applied Biocatalysis, College of Light Industry and Food Sciences, South China University of Technology, Guangzhou, 510640, China

## Abstract

**Background:**

Biocatalytic asymmetric reductions with whole cells can offer high enantioselectivity, environmentally benign processes and energy-effective operations and thus are of great interest. The application of whole cell-mediated bioreduction is often restricted if substrate and product have low water solubility and/or high toxicity to the biocatalyst. Many studies have shown that a biphasic system is often useful in this instance. Hence, we developed efficient biphasic reaction systems with biocompatible water-immiscible ionic liquids (ILs), to improve the biocatalytic anti-Prelog enantioselective reduction of acetyltrimethylsilane (ATMS) to (*R*)-1-trimethylsilylethanol {(*R*)-1-TMSE}, which is key synthon for a large number of silicon-containing drugs, using immobilized *Candida parapsilosis* CCTCC M203011 cells as the biocatalyst.

**Results:**

It was found that the substrate ATMS and the product 1-TMSE exerted pronounced toxicity to immobilized *Candida parapsilosis* CCTCC M203011 cells. The biocompatible water-immiscible ILs can be applied as a substrate reservoir and *in situ* extractant for the product, thus greatly enhancing the efficiency of the biocatalytic process and the operational stability of the cells as compared to the IL-free aqueous system. Various ILs exerted significant but different effects on the bioreduction and the performances of biocatalysts were closely related to the kinds and combination of cation and anion of ILs. Among all the water-immiscible ILs investigated, the best results were observed in 1-butyl-3-methylimidazolium hexafluorophosphate (C_4_mim·PF_6_)/buffer biphasic system. Furthermore, it was shown that the optimum substrate concentration, volume ratio of buffer to IL, buffer pH, reaction temperature and shaking rate for the bioreduction were 120 mM, 8/1 (v/v), 6.0, 30°C and 180 r/min, respectively. Under these optimized conditions, the initial reaction rate, the maximum yield and the product *e.e.* were 8.1 *μ*mol/min g_cwm_, 98.6% and >99%, respectively. The efficient whole-cell biocatalytic process was shown to be feasible on a 450-mL scale. Moreover, the immobilized cells remained around 87% of their initial activity even after being used repeatedly for 8 batches in the C_4_mim·PF_6_/buffer biphasic system, exhibiting excellent operational stability.

**Conclusions:**

For the first time, we have successfully utilized immobilized *Candida parapsilosis* CCTCC M203011 cells, for efficiently catalyzing anti-Prelog enantioselective reduction of ATMS to enantiopure (*R*)-1-TMSE in the C_4_mim·PF_6_/buffer biphasic system. The substantially improved biocatalytic process appears to be effective and competitive on a preparative scale.

## Background

Enantiopure chiral alcohols have been shown to be versatile chiral building blocks for the synthesis of chiral pharmaceuticals, agrochemicals, pheromones, flavors and liquid crystals [1,2]. Nowadays, enantiopure silicon-containing chiral alcohols are becoming increasingly attractive due to the unique physical and chemical characteristics of the silicon atom, such as its larger atomic radius and smaller electronegativity than the carbon atom [3]. Accordingly, these silicon-containing compounds play an important role not only in asymmetric synthesis and functional materials, but also in the preparation of silicon-containing drugs, such as Zifrosilone, Cisobitan and TAC-101{4-[3,5-bis(trimethylsilyl)benzamido] benzoic acid}, which possess greater pharmaceutical activity, higher selectivity and lower toxicity than their carbon counterparts [4-6]. The economic preparation of enantiopure chiral alcohols through asymmetric reduction of prochiral ketones has been proved to be a reliable, scalable and straightforward route by a number of studies [7-9]. When compared to conventional chemical methods, biocatalytic asymmetric reductions using isolated enzymes or whole cells as biocatalyst, can offer high enantioselectivity, environmentally benign processes and energy-effective operations and thus of great interest [10]. The major advantages of using whole cells rather than isolated enzymes as biocatalysts are that cells provide a natural environment for the enzymes, preventing conformational changes in the protein structure that would lead to loss of activity in non-conventional medium, and are able to efficiently regenerate the expensive cofactors [11].

In our previous study, the use of *Saccharomyces cerevisiae* for asymmetric reduction of acetyltrimethylsilane (ATMS) to (*S*)-1-trimethylsilylethanol {(*S*)-1-TMSE} yielded improved results in an aqueous/organic biphasic system as compared to those achieved in a monophase aqueous system [12]. However, use of conventional organic solvents in such processes may be problematic because in many cases they are toxic to the microbial cells and lead to poor operational stability. Also, they may be explosive and are usually environmentally harmful. Ionic liquids (ILs), have recently emerged as novel green solvents for a great variety of biocatalytic transformations, and are becoming more and more attractive in such applications as a promising alternative to the conventional organic solvents [13-16]. Many kinds of ILs have proven to be biocompatible with diverse microbial cells, and present many advantages for the biotransformations such as high conversion rates, high enantioselectivity, excellent operational stability and recyclability [17]. Recently, we have reported the successful synthesis of enantiopure (*S*)-1-TMSE with immobilized *Saccharomyces cerevisiae* cells by using an IL as reaction medium with markedly improved results (yield: 99.2%, product *e.e.* > 99.9%) [18]. To the best of our knowledge, although the biocatalytic reduction of ATMS to (*S*)-1-TMSE has been successfully established following the Prelog rule, the biocatalytic anti-Prelog enantioselective reduction of ATMS to (*R*)-1-trimethylsilylethanol {(*R*)-1-TMSE} using microbial cells, especially in IL-containing systems, has so far remained unexplored, with the exception of only one account we reported where the biotransformation was carried out only in the neat aqueous monophasic system [19]. The biocatalyst used in our previous study was the *Candida parapsilosis* CCTCC M203011 cells, which are capable of effectively catalyzing anti-Prelog stereoselective reduction of a number of carbonyl compounds, maybe due to the possession of four novel anti-Prelog stereoselective carbonyl reductases [20-22]. However, the substrate and the product showed the pronounced inhibitory and toxic effects on the microbial cells in the aqueous monophasic system, thus resulting in relatively lower reactant concentration and reaction efficiency [19].

**Figure 1:**
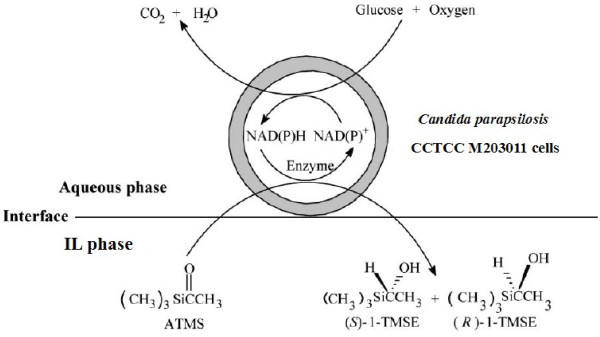
**The bioreduction of ATMS to (*R*)-1-TMSE with immobilized *Candida parapsilosis *CCTCC M203011 cells in water-immiscible IL/buffer biphasic systems.**

In the present study, we, for the first time, report the utilization of various water-immiscible ILs (Table 1) in a two-phase system to efficiently improve the biocatalytic reduction of ATMS to (*R*)-1-TMSE with immobilized *Candida parapsilosis* CCTCC M203011 cells (Figure 1), and the examination of the effect of these ILs on the biocatalytic reaction. In this process, ATMS is reduced to enantiopure (*R*)-1-TMSE while converting NAD(P)H to NAD(P)^+^, and glucose is simultaneously oxidized to CO_2_, presumably driving the reduction reaction by regenerating NAD(P)H from NAD(P)^+^. Moreover, the IL-based biphasic systems can efficiently overcome the limitation of substrate and/or product inhibition often observed during the bioreduction of carbonyl compounds in a monophasic system [23,24], and consequently a high product yield can be achieved without cofactor supplements. Additionally, the efficient biocatalytic process in the presence of ILs was tested on a preparative scale and shown to be effective and competitive.

**Table 1:** Water-immiscible ILs used for the biocatalytic reduction of ATMS and their abbreviations

**Ionic liquid**	**Structure**	**Abbreviation**
1-butyl-3-methylimidazolium hexafluorophosphate		C_4_mim·PF_6_
1-pentyl-3-methylimidazolium hexafluorophosphate		C_5_mim·PF_6_
1-hexyl-3-methylimidazolium hexafluorophosphate		C_6_mim·PF_6_
1-heptyl-3-methylimidazolium hexafluorophosphate		C_7_mim·PF_6_
1-*iso*butyl-3-methylimidazolium hexafluorophosphate		*i*C_4_mim·PF_6_
1-ethyl-3-methylimidazolium bis(trifluoromethanesulfonyl)imide		C_2_mim·Tf_2_N
1-butyl-3-methylimidazolium bis(trifluoromethanesulfonyl)imide		C_4_mim·Tf_2_N
1-hexyl-3-methylimidazolium bis(trifluoromethanesulfonyl)imide		C_6_mim·Tf_2_N
N-butyl-N-methylpyrrolidinium bis(trifluoromethanesulfonyl)imide		C_4_mpyrr·Tf_2_N
N-butyl-N-methylpiperidinium bis(trifluoromethanesulfonyl)imide		C_4_mpip·Tf_2_N
Hexyltributylphosphonium bis(trifluoromethanesulfonyl)imide		P_6,4,4,4_·Tf_2_N

## Results and discussion

### Effect of various water-immiscible ILs on the anti-Prelog enantioselective reduction of ATMS to (*R*)-1-TMSE with immobilized *Candida parapsilosis* CCTCC M203011

**Figure 2:**
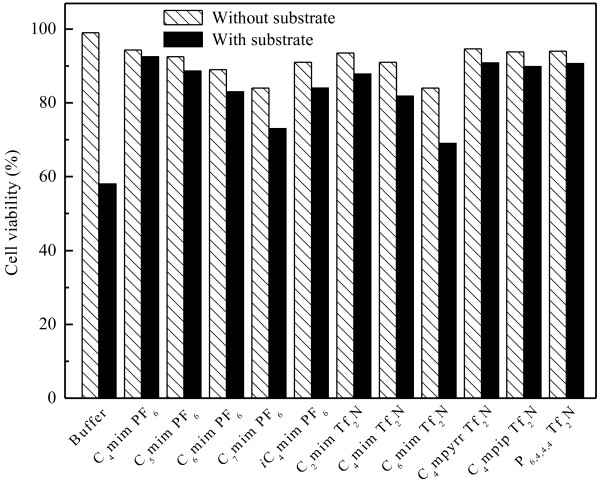
**Cell viability of immobilized *Candida parapsilosis *CCTCC M203011 after exposure for 12 h to various IL-containing biphasic systems.**

**Figure 3:**
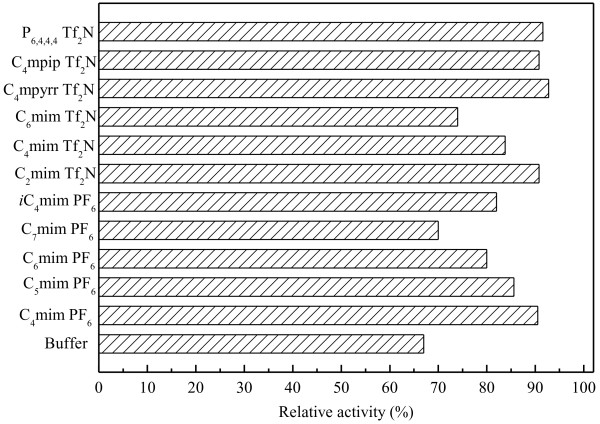
**Deactivation of immobilized *Candida parapsilosis *CCTCC M203011 cell in the presence of 1-TMSE product (40 mM) in various IL-based biphasic systems.** In each case, the 100% relative activity corresponded to the original activity of the cells.

Many studies have shown that a biphasic system is often useful in whole-cell biocatalysis if substrate and product have low water solubility or high toxicity to the biocatalyst [10,25,26]. Therefore, the cell viability of immobilized *Candida parapsilosis* CCTCC M203011 with and without the addition of substrate ATMS were studied in the aqueous monophasic system as well as the IL-based biphasic systems. As shown in Figure 2, the cell viability clearly decreased in the presence of substrate compared to in the absence of substrate in all reaction systems, especially in the aqueous monophasic system, suggesting that ATMS manifests substantial toxicity to immobilized *Candida parapsilosis* CCTCC M203011 cells. It was noted that in the presence of substrate, the cell viability was significantly higher in all the IL-based biphasic systems than in the aqueous monophasic system. Meanwhile, in the absence of substrate, the cell viability was lower in all tested IL-based biphasic systems, compared to the aqueous monophasic system. This indicates that the ILs were toxic to the cells to some extent. Furthermore, to better understand the toxic or inhibitory effects of the product, the deactivation profiles of the cells in different reaction systems in the presence of 40 mM 1-TMSE were investigated (Figure 3). After incubation in the aqueous system with 1-TMSE for 12 h, the cells retained only 67% of their original activity, clearly showing the severe toxic or inhibitory effect of the product. However, the cells in IL-based biphasic systems retained much higher relative activity (as compared to the cells in the aqueous monophasic system) after incubation for a same period. Based on the results depicted in Figure 2 and Figure 3, the water immiscible ILs can be applied as an excellent second liquid phase in the bioreduction process, acting as a substrate reservoir and *in situ* extractant for the product.

Partition coefficients have been applied as an important criterion for the preliminary screening of the second phase. Higher partition coefficients between IL and buffer could effectively reduce the effect of toxic substrate and the product on the cells as well as the pronounced inhibitions of the reaction by the substrate and the product in aqueous monophasic system [25,27]. As demonstrated in Table 2, all the tested ILs possessed considerably high partition coefficients of ATMS and 1-TMSE between IL phase and buffer phase. It was noted that the partition coefficients of ATMS were significantly higher than that of 1-TMSE, due to the stronger lipophilic property of the former. Moreover, combined with the results in Table 2, Figure 2 and Figure 3, higher partition coefficient of substrate and product between two phases correlated with higher biocompatibility of IL with the biocatalysts, which furthermore has an outstanding effect on the overall process efficiency.

**Table 2:** Partition coefficients of ATMS and 1-TMSE between the two phases

**Biphasic system**	**Partition coefficients**	
	**ATMS**	**1-TMSE**
C_4_mim·PF_6_/buffer	55.3	9.6
C_5_mim·PF_6_/buffer	53.9	8.9
C_6_mim·PF_6_/buffer	52.7	8.1
C_7_mim·PF_6_/buffer	50.6	7.6
*i*C_4_mim·PF_6_/buffer	52.8	8.6
C_2_mim·Tf_2_N/buffer	52.8	11.8
C_4_mim·Tf_2_N/buffer	51.3	10.6
C_6_mim·Tf_2_N/buffer	49.1	9.8
C_4_mpyrr·Tf_2_N/buffer	53.5	13.8
C_4_mpip·Tf_2_N/buffer	52.7	11.3
P_6,4,4,4_·Tf_2_N/buffer	53.0	12.5

It has been well known that the influences of different ILs on a biocatalytic reaction mediated by different microorganisms have been found to vary widely, where the performances of biocatalysts were closely related to the kinds and combination of cation and anion of ILs [25,28-30]. Therefore, a comparative study of the effect of ILs with different combination of cation and anion on the bioreduction of ATMS with immobilized *Candida parapsilosis* CCTCC M203011 cells was carried out in various water-immiscible ILs-based biphasic systems (Table 3). It was noted that the immobilized *Candida parapsilosis* CCTCC M203011 cells were capable of efficient synthesis of (*R*)-1-TMSE by anti-Prelog reduction of ATMS in various water-immiscible ILs-based biphasic systems with a high product *e.e.* of above 99%. In general, the specific reaction rate (0.55-1.25 *μ*mol/min g_cwm_) and the maximum yield (78.5-95.8%) in the IL-based biphasic systems were much higher than that in the aqueous monophasic system (0.42 *μ*mol/min g_cwm_ and 70.5%, respectively) under the same reaction conditions.

**Table 3:** **Effect of various water-immiscible ILs on anti-Prelog reduction of ATMS to (*R*)-1-TMSE with immobilized *Candida parapsilosis *CCTCC M203011 cells**

**Medium**	***V***_**o**_^***a***^**(*μ*mol/min g**_**cwm**_**)**	**Yield**^***b***^**(%)**	**Product *e.e. *(%)**
Aqueous buffer	0.42	70.5	>99
C_4_mim·PF_6_/buffer	1.25	95.8	>99
C_5_mim·PF_6_/buffer	0.98	93.1	>99
C_6_mim·PF_6_/buffer	0.78	88.1	>99
C_7_mim·PF_6_/buffer	0.63	81.7	>99
*i*C_4_mim·PF_6_/buffer	0.93	92.2	>99
C_2_mim·Tf_2_N/buffer	0.88	91.9	>99
C_4_mim·Tf_2_N/buffer	0.76	86.7	>99
C_6_mim·Tf_2_N/buffer	0.55	78.5	>99
C_4_mpyrr·Tf_2_N/buffer	0.95	93.2	>99
C_4_mpip·Tf_2_N/buffer	0.88	92.3	>99
P_6,4,4,4_·Tf_2_N/buffer	0.91	92.7	>99

Reaction conditions: 40 mM ATMS, TEA-HCl buffer (100 mM, pH 6.5)/IL volume ratio of 2/1, 20% (w/v) glucose, 0.2 g/mL cell-loaded alginate beads, 30°C, 180 r/min.

^*a*^ Initial reaction rate (*V*_o_) is defined as the initial rate of the product formation in the total reaction system, expressed as the specific activity in μmol product per min per gram of cell wet mass (cwm) unless specified otherwise.

^*b*^ Maximum yield.

In the case of C_n_mim·PF_6_/buffer (n = 4–7) biphasic systems, the initial reaction rate and the maximum yield obviously decreased with the elongation of the alkyl chain attached to the cation (*i.e.* increasing n value) (Table 3). These results were possibly caused by the increase in viscosity of the IL with increasing n value, which may lead to a decrease of substrate and product mass transfer rate between the two phases [13,31,32]. Additionally, both the slightly reduced partition coefficients of ATMS between IL and buffer (Table 2) and the lowered biocompatibility of IL with immobilized *Candida parapsilosis* CCTCC M203011 cells (Figure 2 and Figure 3) with increasing n value could lead to this phenomenon. When the *n*-butyl group attached to the imidazolium cation of C_4_mim·PF_6_ was replaced by *iso*-butyl (*i*C_4_MIM·PF_6_), the initial reaction rate and the maximum yield obviously declined, indicating that a minor change of IL structure exerts a substantial influence on the catalyst performance. Coincidently, a decrease in cell viability and partition coefficients (Figure 2, Figure 3 and Table 1) was observed with the variation of IL structure from C_4_MIM·PF_6_ to *i*C_4_MIM·PF_6_. For the C_n_mim·Tf_2_N/buffer (n = 2, 4, 6) biphasic systems, the change profiles of the initial reaction rate and the maximum yield with the elongation of the alkyl chain agree with those observed in the C_n_mim·PF_6_/buffer (n = 4–7) media (Table 3). In the last three Tf_2_N^−^-based (C_4_mpyrr·Tf_2_N, C_4_mpip·Tf_2_N and P_6,4,4,4_·Tf_2_N) biphasic systems, the bioreduction efficiency was different with change of IL cation.

**Figure 4:**
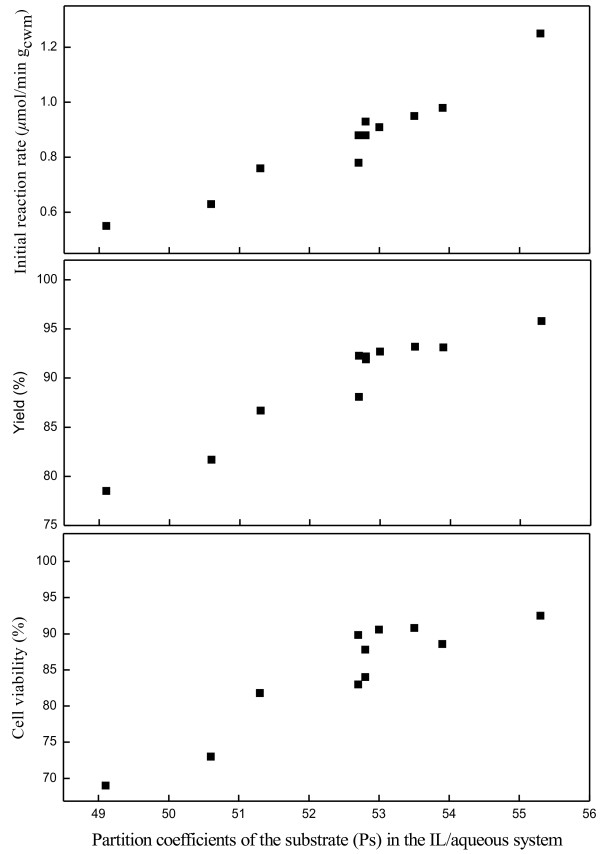
Effect of partition coefficients of the substrate (Ps) on the initial reaction rate, yield and cell viability.

On the whole, the diverse ILs showed significant but different effects on the catalytic performance of immobilized *Candida parapsilosis* CCTCC M203011 cells and the bioreduction reaction. And it was interesting to found that the initial reaction rates, yields and cell viability are tightly associated with the partition coefficients of the substrate (Ps) in the IL/aqueous system. Indeed, approximate linear relationship could be obtained by drawing initial reaction rates, yields and cell viability against Ps, respectively (Figure 4). As illustrated in Figure 4, the initial reaction rates, yields and cell viability all displayed an increase trend with the rise of Ps, which indicated that the ILs affect the activity of biocatalyst by influencing the substrate concentration in the aqueous layer around the biocatalyst to a great extent. Therefore, the superior performance of the biocatalyst found in the IL-containing system could be attributed to the markedly reduced effect of toxic substrate on the cells and substrate inhibition, since the ILs could effectively extract substrate from the aqueous phase. This finding was consistent with that of Yang and Robb, in which Ps gave systematic relations with activity of mushroom tyrosinase in organic solvent/aqueous system [33].

Based on the above results, among all the water-immiscible ILs investigated, C_4_mim·PF_6_ gave the fastest initial reaction rate and the highest yield. It should be noted that the common ILs holding PF_6_ as the anion are concerned for their potential hazards because their hydrolysis may induce the release of HF [34]. In our study, the best bioreduction results and excellent cell biocompatibility were observed in C_4_mim·PF_6_/buffer biphasic system, which was consistent with many other published papers [10,25,35]. Hence, C_4_mim·PF_6_ was chosen as the best second phase in IL/buffer biphasic system for subsequent experiments and meanwhile several new types of water-immiscible ILs which are believed to be safer than the present one are underway for the improvement of this bioreduction process.

### Effects of key variables on the biocatalytic reduction of ATMS

For a better understanding of the biocatalytic anti-Prelog reduction of ATMS to (*R*)-1-TMSE with immobilized *Candida parapsilosis* CCTCC M203011 cells performed in the C_4_mim·PF_6_/buffer biphasic system, the effects of several crucial variables such as substrate concentration, volume ratio of buffer to IL, buffer pH, reaction temperature and shaking rate were studied systematically.

**Figure 5:**
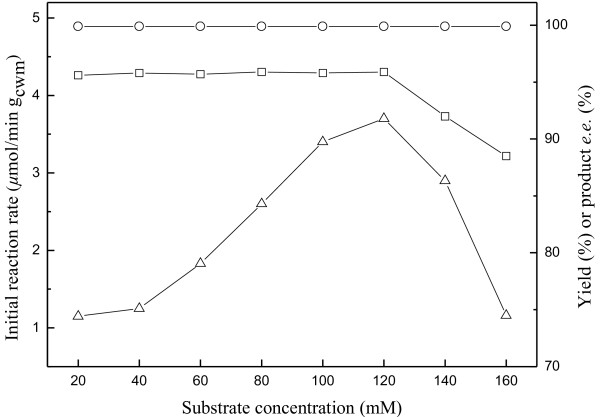
**Effect of substrate concentration on the bioreduction of ATMS catalyzed by immobilized *Candida parapsilosis *CCTCC M203011 cells.** Symbols: (△) the initial reaction rate; (□) the maximum product yield of GC analysis; (○) the product *e.e.* All products have the (*R*) configuration. Reaction conditions: various concentrations of ATMS, TEA-HCl buffer (100 mM, pH 6.5)/IL volume ratio of 2/1, 20% (w/v) glucose, 0.2 g/mL cell-loaded alginate beads, 30°C, 180 r/min.

As shown in Figure 5, the initial reaction rate increased with increasing substrate concentration up to 120 mM, while the maximum yield and product *e.e.* showed no clear variation. However, further increase in substrate concentration led to a marked drop in the initial reaction rate and the maximum yield, possibly caused by the substrate inhibition at high concentrations of substrate. Obviously, the optimal substrate concentration in the C_4_mim·PF_6_/buffer biphasic system was 120 mM, which was much higher than that in the aqueous monophasic system (20 mM) [19].

**Figure 6:**
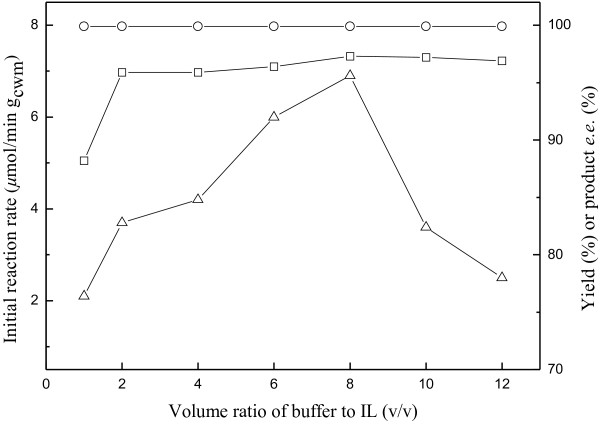
**Effect of volume ratio of buffer to IL on the bioreduction of ATMS catalyzed by immobilized *Candida parapsilosis *CCTCC M203011 cells.** Symbols: (△) the initial reaction rate; (□) the maximum product yield of GC analysis; (○) the product *e.e.* All products have the (*R*) configuration. Reaction conditions: 120 mM ATMS, various volume ratio of TEA-HCl buffer (100 mM, pH 6.5)/IL, 20% (w/v) glucose, 0.2 g/mL cell-loaded alginate beads, 30°C, 180 r/min.

It has been well known that the volume ratio of two phases exerts a great impact on biocatalytic reactions with whole cells in biphasic systems, influencing not only the interfacial areas but also the viability of microbial cells [36,37]. As illustrated in Figure 6, the volume ratio of the aqueous buffer phase to the IL phase (V_aq_/V_IL_, mL/mL) substantially affected the initial reaction rate and the maximum yield, but had little effect on the product *e.e*. An obvious enhancement in the initial reaction rate and the maximum yield was observed with the increase of V_aq_/V_IL_ up to 8/1, possibly owing to the less chance for the cells to contact with substrate molecules dissolved in IL, thus reducing the inactivation effect of substrate and/or IL on biocatalyst. Further rise in the V_aq_/V_IL_ ratio resulted in a decrease in the initial reaction rate, which may be attributed to the lower substrate concentration in the aqueous phase. So it is clear that 8/1 is the optimum V_aq_/V_IL_ ratio for the reaction.

**Figure 7:**
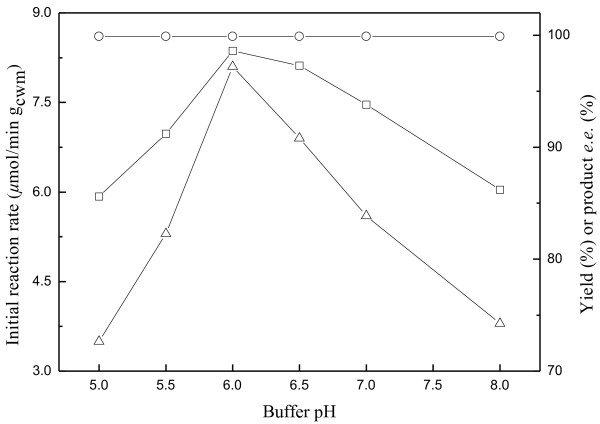
**Effect of buffer pH on the bioreduction of ATMS catalyzed by immobilized *Candida parapsilosis *CCTCC M203011 cells.** Symbols: (△) the initial reaction rate; (□) the maximum product yield of GC analysis; (○) the product *e.e.* All products have the (*R*) configuration. Reaction conditions: 120 mM ATMS, TEA-HCl buffer (100 mM, various pH)/IL volume ratio of 8/1, 20% (w/v) glucose, 0.2 g/mL cell-loaded alginate beads, 30°C, 180 r/min.

Buffer pH is one of the most important parameters affecting enzyme-catalyzed reactions [23,38,39]. The great impact of buffer pH on the bioreduction of ATMS in C_4_mim·PF_6_/buffer biphasic system was exhibited in Figure 7. Increasing buffer pH from 5.0 to 6.0 gave rise to an increase in the initial reaction rate from 3.5 *μ*mol/min g_cwm_ to 8.1 *μ*mol/min g_cwm_, while the maximum yield markedly increased from 85.6% to 98.6%. However, further increase in the buffer pH led to a clear drop in the initial reaction rate and the maximum product yield. Moreover, buffer pH showed little influence on the product *e.e.* and kept above 99.9% within the range tested, indicating that there was no problem with activity of undesired isoenzymes within this range of pH. Based on these results, it is clear that pH 6.0 is the optimal value for the bioreduction.

**Figure 8:**
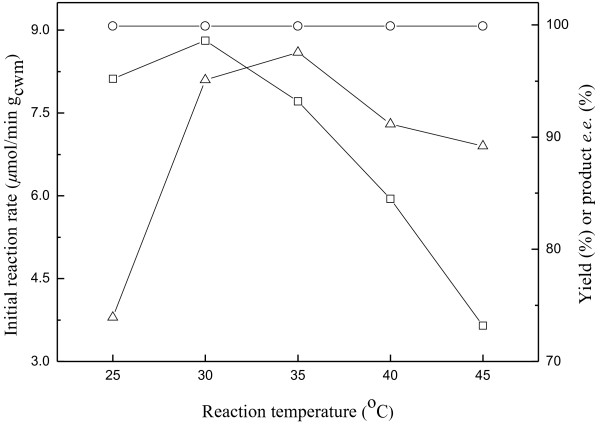
**Effect of reaction temperature on the bioreduction of ATMS catalyzed by immobilized *Candida parapsilosis *CCTCC M203011 cells.** Symbols: (△) the initial reaction rate; (□) the maximum product yield of GC analysis; (○) the product *e.e.* All products have the (*R*) configuration. Reaction conditions: 120 mM ATMS, TEA-HCl buffer (100 mM, pH 6.0)/IL volume ratio of 8/1, 20% (w/v) glucose, 0.2 g/mL cell-loaded alginate beads, various reaction temperatures, 180 r/min.

Reaction temperature has a crucial impact on the activity, selectivity and stability of a biocatalyst and the equilibrium of a reaction as well [23,39]. As exhibited in Figure 8, the initial reaction rate markedly boosted with increasing reaction temperature from 25°C to 35°C, since higher temperatures may accelerate the molecular collisions between the enzyme and substrate. However, the maximum product yield sharply decreased when the temperature was above 30°C, which might be due to the partial inactivation of the enzyme in the whole cells at a higher temperature. Throughout the tested range of reaction temperature, the product *e.e.* showed no variation and kept above 99%. Therefore, the suitable reaction temperature for the bioreduction was 30°C.

**Figure 9:**
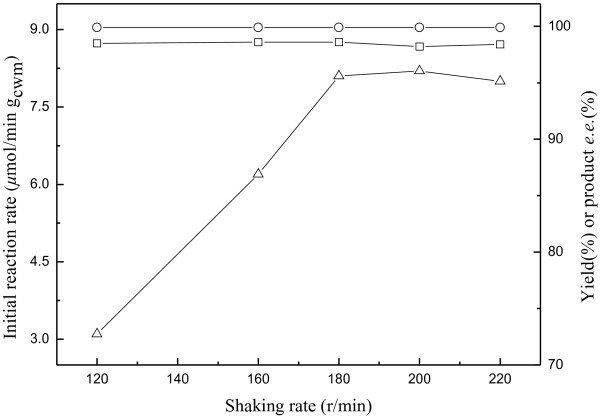
**Effect of shaking rate on the bioreduction of ATMS catalyzed by immobilized *Candida parapsilosis *CCTCC M203011 cells.** Symbols: (△) the initial reaction rate; (□) the maximum product yield of GC analysis; (○) the product *e.e.* All products have the (*R*) configuration. Reaction conditions: 120 mM ATMS, TEA-HCl buffer (100 mM, pH 6.0)/IL volume ratio of 8/1, 20% (w/v) glucose, 0.2 g/mL cell-loaded alginate beads, 30°C, various shaking rates.

Shaking rate affects the diffusion and partition of substrate and the product in the biphasic reaction system and thus leads to the changes in the initial reaction rate, the maximum yield and product *e.e.*, especially in IL-containing systems. High viscosity of IL limits the diffusion of substrates and products to and from the active site of enzyme [40,41]. As shown in Figure 9, the initial reaction rate increased rapidly with the increase of shaking rate up to 180 r/min, suggesting that the mass transfer was the rate-limiting step. However, further increase in the shaking rate had little effects on the initial reaction rate, the maximum yield and the product *e.e.*, indicating that 180 r/min was the optimal shaking rate for the bioreduction in the C_4_mim·PF_6_/buffer biphasic system.

Under the optimum conditions described above, the efficiency of the biocatalytic anti-Prelog stereoselective reduction of ATMS to (*R*)-1-TMSE with immobilized *Candida parapsilosis* CCTCC M203011 cells was substantially enhanced in the C_4_mim·PF_6_-containing biphasic system compared to the neat aqueous buffer system [19] at each optimum reaction conditions, in terms of optimum substrate concentration (120 mM *vs* 20 mM), initial reaction rate (8.1 *μ*mol/min g_cwm_*vs* 0.98 *μ*mol/min g_cwm_), and maximum yield (98.6% *vs* 96.5%). The product *e.e.* still kept above 99%. When the substrate concentration exceeded 20 mM, the initial reaction and the maximum yield decreased rapidly in the neat aqueous system because of the inhibitory and toxic effect of substrate and product [19]. Therefore, the use of C_4_mim·PF_6_/buffer biphasic system, instead of aqueous monophasic system, can markedly improve the biocatalytic reduction of ATMS.

### Operational stability of immobilized Candida parapsilosis CCTCC M203011 cells

**Figure 10:**
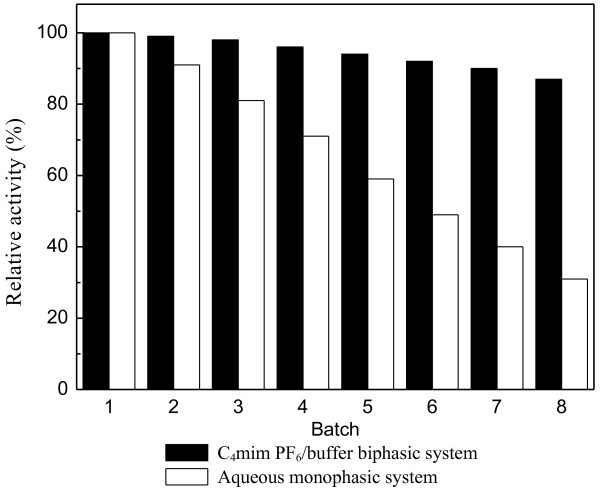
**Operational stability of immobilized *Candida parapsilosis *CCTCC M203011 cells.** Reaction conditions with C_4_mim·PF_6_/buffer biphasic system: 120 mM ATMS, C_4_mim·PF_6_/TEA-HCl buffer (100 mM, pH 6.0) volume ratio of 1/8, 20% (w/v) glucose, 0.2 g/mL cell-loaded alginate beads, 30°C, 180 r/min, 12 h per batch. Reaction conditions with aqueous system: 20 mM ATMS, 4.5 mL of TEA-HCl buffer (100 mM, pH 6.0), 20% (w/v) glucose, 0.2 g/mL cell-loaded alginate beads, 30°C, 180 r/min, 12 h per batch. The relative activity of the immobilized cells in the first batch was defined as 100%.

For a deeper understanding on the effect of IL on the whole cell bioreduction, it is essential to make a comparative study on the operational stability of the immobilized *Candida parapsilosis* CCTCC M203011 cells in different systems. As shown in Figure 10, the operational stability of the immobilized cells was significantly enhanced in the C_4_mim·PF_6_/buffer biphasic system as compared to that in the aqueous monophasic system. The immobilized cells still remained around 87% of their original activity even after being used repeatedly for 8 batches in the C_4_mim·PF_6_/buffer biphasic system. In contrast, the relative activity of the immobilized cells was only about 31% after being re-used for the same operational batches in the aqueous monophasic system. The good biocompatibility of the IL C_4_mim·PF_6_ and its excellent solvent properties for the toxic substrate and product could partly account for the observations. The improved interactions between the IL and the carrier (calcium alginate) [42] used for the immobilization of *Candida parapsilosis* CCTCC M203011 cells may also result in the good operational stability of the immobilized cells in the C_4_mim·PF_6_-containing system. The cells maybe also become coated with the IL and thus protected from the inactivation.

### Preparative scale biotransformation in the C_4_mim·PF_6_-based biphasic system

To show the applicability of the biocatalytic anti-Prelog reduction of ATMS to (*R*)-1-TMSE using immobilized *Candida parapsilosis* CCTCC M203011 cells in the C_4_mim·PF_6_/buffer biphasic system, we also performed the bioreduction on a 450-mL preparative scale under the optimized reaction conditions detailed above. The reaction process was monitored by GC analysis and the product was extracted from the reaction mixture with *n*-hexane upon the completion of the reaction. The bioreduction behavior was similar to that shown on the 4.5-mL experimental scale. In spite of being marginally lower than that obtained on the 4.5-mL scale, the isolated yield of (*R*)-1-TMSE (97.5%) after 12 h reaction on the 450-mL scale was much higher than that in the neat aqueous system at the identical reaction conditions, and the product *e.e.* was more than 99%. Furthermore, no emulsification of the IL-based biphasic system was found, so the phases could be separated readily by centrifugation. The IL could also be easily recycled, lowering the overall cost of the biocatalytic process. Hence, the biocatalytic reduction of ATMS in the presence of C_4_mim·PF_6_ exhibited an enormous potential of industrial application.

## Conclusions

The efficient synthesis of (*R*)-1-TMSE can be successfully conducted with high yield and excellent product *e.e. via* the biocatalytic anti-Prelog enantioselective reduction of ATMS using immobilized *Candida parapsilosis* CCTCC M203011 cells in a two-phase system containing water-immiscible ILs. The diverse ILs with different combination of cation and anion, different length of the alkyl chain attached to the cation and different partition coefficients of the substrate in the IL/aqueous system all showed significant but different effects on the catalytic performance of immobilized *Candida parapsilosis* CCTCC M203011 cells and the bioreduction reaction. Among the examined ILs, the IL C_4_mim·PF_6_ boosted markedly the reaction efficiency of the bioreduction and gave the best biotransformation results, which may be due to the IL’s excellent solvent property for substrate and its good biocompatibility with the microbial cells. Also, the immobilized cells manifested very good operational stability in the presence of C_4_mim·PF_6,_ as supported by the observation that it still maintained a much higher activity in the C_4_mim·PF_6_-based system than that in the neat aqueous system after successive re-use for 8 batches (87% *vs* 31%). Moreover, the results described here clearly show that the whole-cell biocatalytic process with C_4_mim·PF_6_ is promising for efficient synthesis of (*R*)-1-TMSE and feasible up to a 450-mL preparative scale.

## Methods

### Biological and chemical materials

*Candida parapsilosis* CCTCC M203011 from the China Center for Type Culture Collection (CCTCC, Wuhan, China) was kindly donated by Professor Yan Xu (Key Laboratory of Industrial Biotechnology, Ministry of Education, School of Biotechnology, Jiangnan University, China).

Acetyltrimethylsilane (ATMS, 97% purity), (*S*)-1-trimethylsilylethanol {(*S*)-1-TMSE, 98% purity}, (*R*)-1-trimethylsilylethanol {(*R*)-1-TMSE, 98% purity} and *n*-nonane (>99% purity) were purchased from Aldrich-Fluka (USA). The eleven ILs shown in Table 1 were purchased from Lanzhou Institute of Chemical Physics (China) and were all of over 98% purity. All other chemicals were also from commercial sources and were of analytical grade.

### Cultivation and immobilization of *Candida parapsilosis* CCTCC M203011 cells

*Candida parapsilosis* CCTCC M203011 cells were cultivated and then immobilized using calcium alginate entrapment method according to our previous study [43]. In brief, a homogenous cell/sodium alginate suspension was firstly prepared and then added dropwise by an injector to a gently stirred CaCl_2_ solution (2%, w/v), where the calcium alginate beads were precipitated.

### General procedure for the biocatalytic asymmetric reduction of ATMS to (*R*)-1-TMSE

In a typical experiment, the biphasic system (4.5 mL) consisted of a water-immiscible IL and TEA-HCl buffer (100 mM, various pHs: 5.0-8.0), contained in a 20-mL Erlenmeyer flask capped with a septum. Alginate beads were prepared that were loaded with 31% (w/w) *Candida parapsilosis* CCTCC M203011 cells {based on cell wet mass (cwm)}and 0.2 g of these cell-loaded alginate beads were added per mL of the aqueous phase, together with 20% (w/v) glucose. The reaction mixture was pre-incubated in a water-bath shaker at 180 r/min and various temperatures (25-45°C) for 15 min. Then, the reactions were initiated by adding ATMS at various concentrations (20–160 mM, based on the volume of the IL phase). Aliquots (10 *μ*L) were withdrawn at specified time intervals from the IL phase and the aqueous phase, respectively, and the product as well as the residual substrate was extracted with *n*-hexane (50 *μ*L) containing 5.6 mM *n*-nonane (as an internal standard), prior to GC analysis. Details of the IL used, volume ratio of buffer to IL, substrate concentration, buffer pH and reaction temperature are specified for each case.

The preparative scale biocatalytic reduction of ATMS to (*R*)-1-TMSE was carried out by adding 80 g of immobilized *Candida parapsilosis* CCTCC M203011 cells and 6 mmol of ATMS to 450 mL of the biphasic system (volume ratio: 1/8) consisted of C_4_mim·PF_6_ and TEA-HCl buffer (100 mM, pH 6.0) containing 20% (w/v) glucose at 30°C and 180 r/min. The reaction was terminated when no substrate was detectable by GC analysis. The immobilized cells were removed by filtration, and the products were extracted from the reaction mixture with *n*-hexane. The product *e.e.* and the isolated yield were determined by GC analysis.

### GC analysis

Reaction mixtures were analyzed according to the GC analysis method in our previous work [18]. The retention times for ATMS, *n*-nonane, (*R*)-1-TMSE and (*S*)-1-TMSE were 3.53 min, 6.09 min, 6.74 min and 7.35 min, respectively. The average error for this determination was less than 1.0%. All reported data were averages of experiments performed at least in duplicate.

### Cell viability assay

The viability of immobilized *Candida parapsilosis* CCTCC M203011 cells was assayed after incubating the alginate-immobilized cells for 12 h in various biphasic systems consisting of water-immiscible ILs and TEA-HCl buffer (100 mM, pH 6.5) (IL/buffer volume ratio: 1/2), or TEA-HCl buffer (100 mM, pH 6.5) system with and without the addition substrate (40 mM ATMS, based on the volume of the IL phase), respectively. The beads of immobilized *Candida parapsilosis* CCTCC M203011 cells were withdrawn from the reaction systems and then added to 0.1 M trisodium citrate to dissolve the alginate. After this, the microbial cell suspension was diluted and stained with 0.1% Methylene Blue for 5 min. Micrographs were taken and analyzed for blue dead cells and colorless viable ones. The cell viability was expressed as the percentage of viable ones in the total cells and the values were given as mean value ± standard deviation (n = 3).

### Determination of partition coefficients

Partition coefficients (K_IL/aq_) were determined by dissolving 12, 24 or 36 mM ATMS or 1-TMSE, as appropriate, in each IL/buffer biphasic system (IL/buffer volume ratio: 1/2) and shaking (180 r/min) for 36 h at 30°C. The concentrations of ATMS or 1-TMSE in the IL phase and the aqueous phase were then analyzed by GC. The concentration of ATMS or 1-TMSE in each phase varied linearly with the total amount of each chemical added to the two-phase system. Then the slopes were calculated and used for the quantification of the partition coefficients of ATMS and 1-TMSE between the IL phase and the aqueous phase.

### Operational stability of immobilized *Candida parapsilosis* CCTCC M203011 cells

In order to assess the operational stability of the cells, the re-use of the immobilized *Candida parapsilosis* CCTCC M203011 cells was investigated in the C_4_mim·PF_6_/buffer biphasic system and also in the aqueous monophasic system. Initially, aliquots of the cells were added into separate screw-capped vials each containing 4.5 mL of the appropriate medium {C_4_mim·PF_6_/TEA-HCl buffer (100 mM, pH 6.0) biphasic system (volume ratio: 1/8), or aqueous TEA-HCl buffer system (100 mM, pH 6.0)}, together with the optimal amount of ATMS and glucose for the reduction conducted in the various media. Then, the bioreductions were carried out at 30°C and 180 r/min and were repeated over 8 batches without changing the immobilized cells. Between batches, the immobilized cells were filtered off from the reaction mixture, washed twice with fresh water, and added to a fresh batch of reaction medium. The reduction activity of the cells was assayed in each batch. The relative activity of the cells employed for the first batch was defined as 100%.

## Competing interests

The authors declare that they have no competing interests.

## Authors' contributions

WYL and MHZ designed research and analyzed the experiment data; BBZ carried out experiments and wrote the paper; PW and JC carried out experiments and conducted GC analysis. All authors read and approved the final manuscript.
